# Metformin inhibits TGF-β1-induced epithelial-to-mesenchymal transition-like process and stem-like properties in GBM *via* AKT/mTOR/ZEB1 pathway

**DOI:** 10.18632/oncotarget.23317

**Published:** 2017-12-15

**Authors:** Yang Song, Yong Chen, Yunqian Li, Xiaoyan Lyu, Jiayue Cui, Ye Cheng, Liyan Zhao, Gang Zhao

**Affiliations:** ^1^ Department of Neurosurgery, First Hospital, Jilin University, Changchun, Jilin 130021, China; ^2^ Department of Medical Laboratory, Second Hospital, Jilin University, Changchun, Jilin 130041, China; ^3^ Department of Histology and Embryology, College of Basic Medicine, Jilin University, Changchun, Jilin 130021, China

**Keywords:** glioblastoma, metformin, epithelial-to-mesenchymal transition, glioblastoma stem cells, AKT/mTOR/ZEB1 pathway

## Abstract

Glioblastoma (GBM) is the most frequent and aggressive brain tumor in adults. In spite of advances in diagnosis and therapy, the prognosis is still relatively poor. The invasive property of GBM is the major cause of death in patients. Epithelial-to-mesenchymal transition-like process (EMT-like process) is considered to play an important role in the invasive property. Metformin has been reported as a regulator of EMT-like process. In this study, we confirmed that metformin inhibited TGF-β1-induced EMT-like process and EMT-associated migration and invasion in LN18 and U87 GBM cells. Our results also showed that metformin significantly suppressed self-renewal capacity of glioblastoma stem cells (GSCs), and expression of stem cell markers Bmi1, Sox2 and Musashi1, indicating that metformin can inhibit cancer stem-like properties of GBM cells. We further clarified that metformin specifically inhibited TGF-β1 activated AKT, the downstream molecular mTOR and the leading transcription factor ZEB1. Taken together, our data demonstrate that metformin inhibits TGF-β1-induced EMT-like process and cancer stem-like properties in GBM cells *via* AKT/mTOR/ZEB1 pathway and provide evidence of metformin for further clinical investigation targeted GBM.

## INTRODUCTION

Glioma is the most frequently occurring type of primary malignant tumors in human brain [1]. The current standard therapies include maximal safe resection followed by radiotherapy in combination with temozolomide [2]. Even with continuous improvements in the treatment, the prognosis is still poor with a median overall survival of only 14.6 months after diagnosis [3].

Glioblastoma (GBM) is the most aggressive brain tumor and often displays strong invasive properties [4]. The invasive phenotype is key to the clinical progression of malignant glioma [5], complicating complete surgical resection and permitting postoperative tumor regrowth. Therefore, there is a great need for novel mechanistic understanding of tumor invasiveness and migration, which would be critical for developing more effective therapies.

The recent studies have suggested that epithelial-to-mesenchymal transition (EMT) has been pointed as one of the mechanisms that confer to the invasive property of glioma cells [6]. However, EMT phenomenon in glioma cells is currently controversially discussed.

EMT is a fundamental process for morphogenesis during embryonic development, tissue remodeling and wound healing [7, 8]. Recently it has also got more attention in cancer progression, invasiveness and metastasis [9, 10]. Through this EMT process, cells may acquire an invasive phenotype that may contribute to tumor recurrence and metastasis. The current knowledge suggests that cancer stem cells (CSCs) are also responsible for the highly invasive and resistant potential of many human brain tumors [11]. Therefore, EMT and CSCs are urgent problems to be solved.

Transforming growth factor-β (TGF-β) is a potent inducer of EMT and positive regulator of tumor progression and metastasis [12, 13]. TGF-β1 has been shown to activate various downstream pathways including PI3K, Smads, and MAPK, which are involved in TGF-β-induced EMT [14]. However, there is still a lot to investigate about its role to influence EMT-like change in glioma cells.

Metformin (Met) is an oral biguanide used in the clinical management of type 2 diabetes mellitus [15]. In addition to its antidiabetic role, metformin has been reported to depress tumor growth in various cancers, including glioma cell lines [16]. Although the anticancer effects of metformin have been known for many years, little is known about the activity of this drug on EMT.

In the present study, we first demonstrated that TGF-β1 induced EMT-like process and enhanced the migratory and invasive ability of glioblastoma cells. And then, we found that metformin inhibited this EMT-like process and inhibited the generated cancer stem-like properties induced by TGF-β1. Furthermore, we explored the underline mechanism in this special procedure. Our results provide supporting evidence to warrant further clinical trial for metformin as a safe and potent antiglioma drug.

## RESULTS

### TGF-β1 induces an EMT-like process in GBM cells

**Figure 1:**
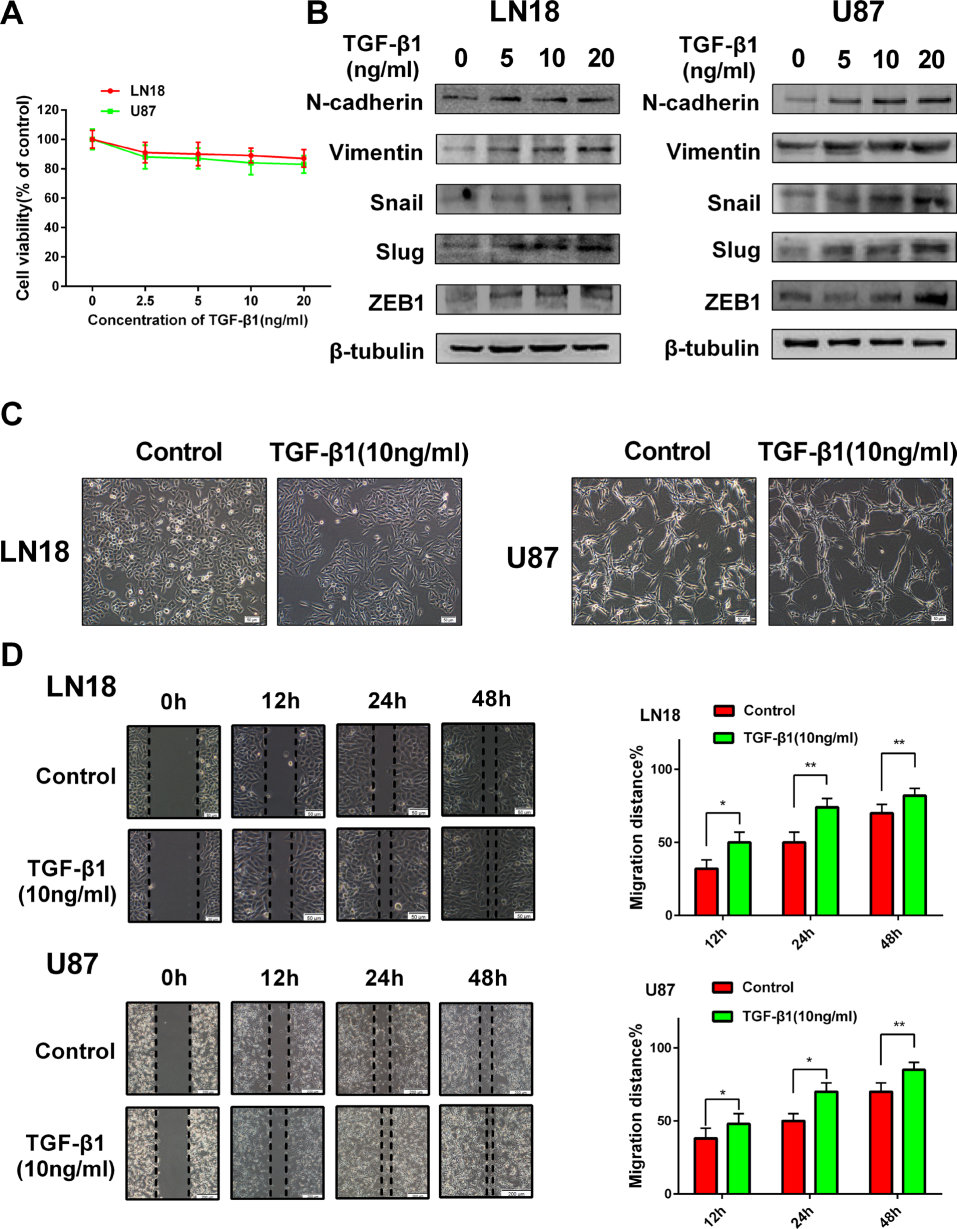
TGF-β1 induces an EMT-like process in GBM cells (**A**) MTT assay of cell viability in LN18 and U87 cells following exposure to TGF-β1 for 48 hours. (**B**) Western blot results of expressions of EMT-related proteins and transcription factors derived from LN18 and U87 cells treated with increasing concentration of TGF-β1 (0, 5, 10 and 20 ng/ml) for 48 hours. (**C**) The morphological changes of LN18 and U87 cells after exposure to TGF-β1 (10 ng/ml) for 48 hours under light microscope (×100 magnification). (**D**) Representative wound-healing images show migratory capacity in LN18 (×100 magnification) and U87 (×40 magnification) cells following exposure to TGF-β1 (10 ng/ml) compared with control group. Histograms show the mean level of migration distance observed in three random fields for each condition. ^*^P < 0.05, ^**^P < 0.01, control group versus TGF-β1 treated group for their respective time points.

To explore the suitable concentration of TGF-β1 in inducing GBM cells, we first examined the cell viability of LN18 and U87 cells in response to TGF-β1 treatment (Figure 1A) and then the concentrations of TGF-β1 were chosen to be used in the following experiments.

GBM cells were hungry in serum-free medium for 12 hours and then increasing concentrations (0, 5, 10 and 20 ng/ml) of TGF-β1 were added to the medium. The cells were continuous cultured for 48 hours. To clarify the EMT-like change in GBM cells, we investigated the expression levels of relative protein markers. We found that N-cadherin and Vimentin expression levels were increased in LN18 and U87 cells in a dose-dependent manner (Figure 1B). Snail, Slug and ZEB1 are reported as vital transcription factors involved in EMT. So, we also examined the expression levels of these factors. As anticipated, Snail, Slug and ZEB1 expression levels were also increased in LN18 and U87 cells in a dose-dependent manner (Figure 1B). We confirmed that 10 ng/ml concentration of TGF-β1 was efficient enough to induce the transition.

Then we examined the effect of TGF-β1 on the morphologic changes of GBM cells. Exposure to TGF-β1 (10 ng/ml) for 48 hours led to a change in cellular morphology that was characterized by a more stretched and elongated appearance and an enhanced scattered growth pattern (Figure 1C).

Next, we examined the effect of TGF-β1-induced EMT-like change on the migration capacity of GBM cells. As anticipated, LN18 and U87 cells treated with TGF-β1 showed enhanced migratory capacity when compared with the untreated control group in wound- healing assays (Figure 1D).

Taken together, these data indicate that TGF-β1 can induce an EMT-like process in GBM cells and promote their migratory potential *in vitro*.

### Metformin inhibits TGF-β1-induced EMT-like process in GBM cells

**Figure 2:**
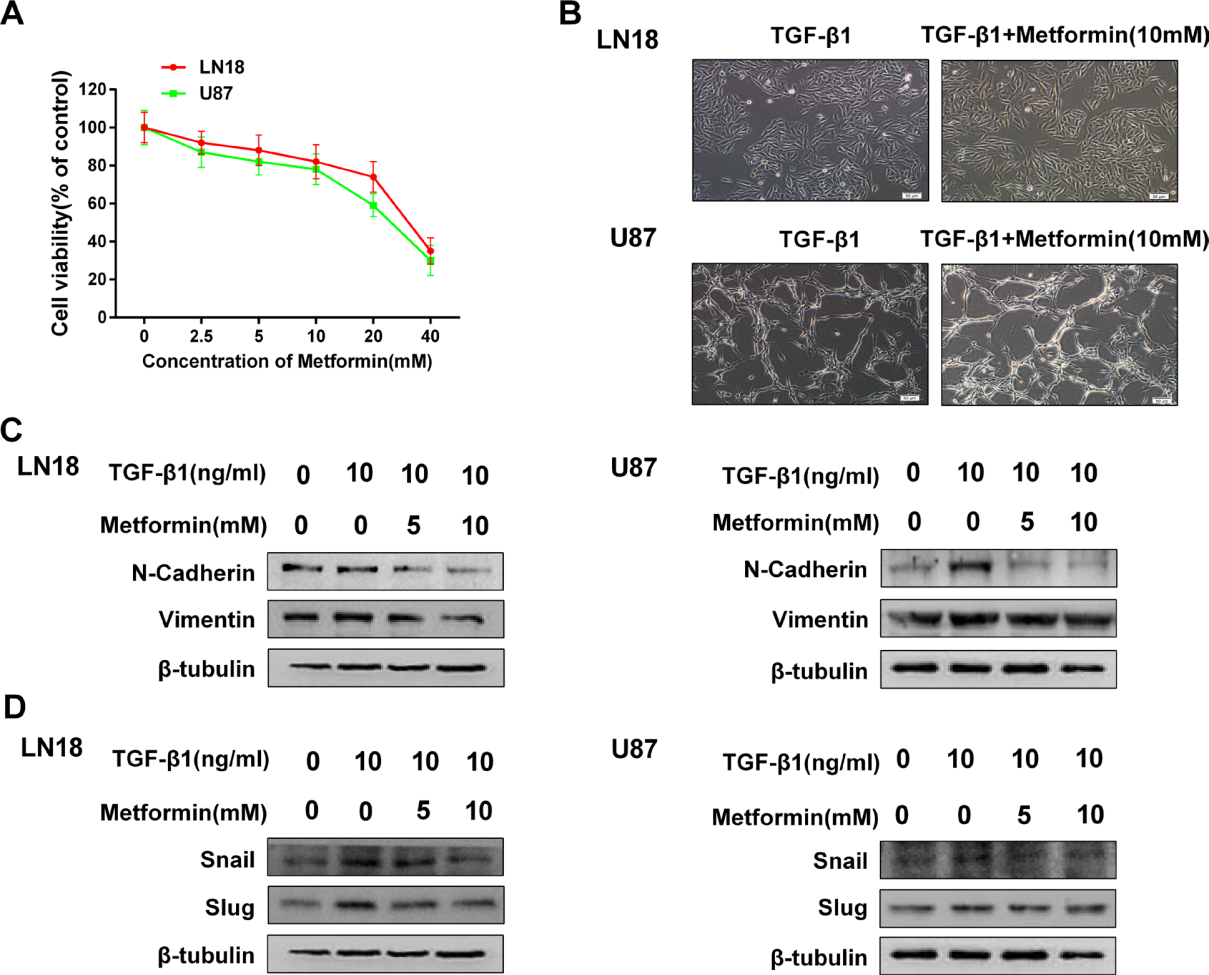
Metformin inhibits TGF-β1-induced EMT-like process in GBM cells (**A**) MTT assay of cell viability in LN18 and U87 cells following exposure to metformin for 48 hours. (**B**) The morphological changes of LN18 and U87 cells after exposure to TGF-β1 (10 ng/ml) with or without metformin for 48 hours under light microscope (×100 magnification). (**C**) Western blot results of dose-dependent inhibition effect of metformin on N-cadherin, Vimentin expression in LN18 and U87 cells. (**D**) Western blot results of dose-dependent inhibition effect of metformin on Snail, Slug expression in LN18 and U87 cells.

We first examined the toxicity of metformin to exclude the influence of cell death or inhibition of proliferation. We found that metformin had little effect on inhibition of proliferation below the concentration of 10 mM (Figure 2A), therefore we chose a concentration range of metformin lower than 10 mM for subsequent studies.

To test whether metformin has an inhibitory effect on EMT-like process in GBM cells, we first observed the morphologic changes following treatment with metformin and TGF-β1 in LN18 and U87 cells compared with treatment with TGF-β1 alone.

As a result, treatment with metformin had little effect on the morphologic changes which caused by treatment with TGF-β1 (Figure 2B), indicating that metformin may not influence the morphology.

To further clarify whether treatment with metformin may influence TGF-β1-induced EMT process, we next examined the expression patterns of mesenchymal marker N-cadherin and Vimentin. We found that metformin down regulated the expression levels of N-cadherin and Vimentin in a dose-dependent manner (Figure 2C). As expected, metformin treatment also significantly suppressed TGF-β1-induced upregulation of Snail, Slug and ZEB1 expression levels in a dose-dependency (Figure 2D). These findings suggest that metformin is able to inhibit TGF-β1-induced EMT-like process in GBM cells by modifying the expression levels of EMT related proteins and transcription factors.

### Metformin suppresses TGF-β1-induced cell migration and invasion

The functional significance of metformin inhibiting the expression profiles of the above EMT-related protein markers and transcription factors was expected to be reflected in the cell migration and invasion. To evaluate the alteration of tumor cell migratory and invasive properties, wound-healing and Transwell assays were performed.

**Figure 3:**
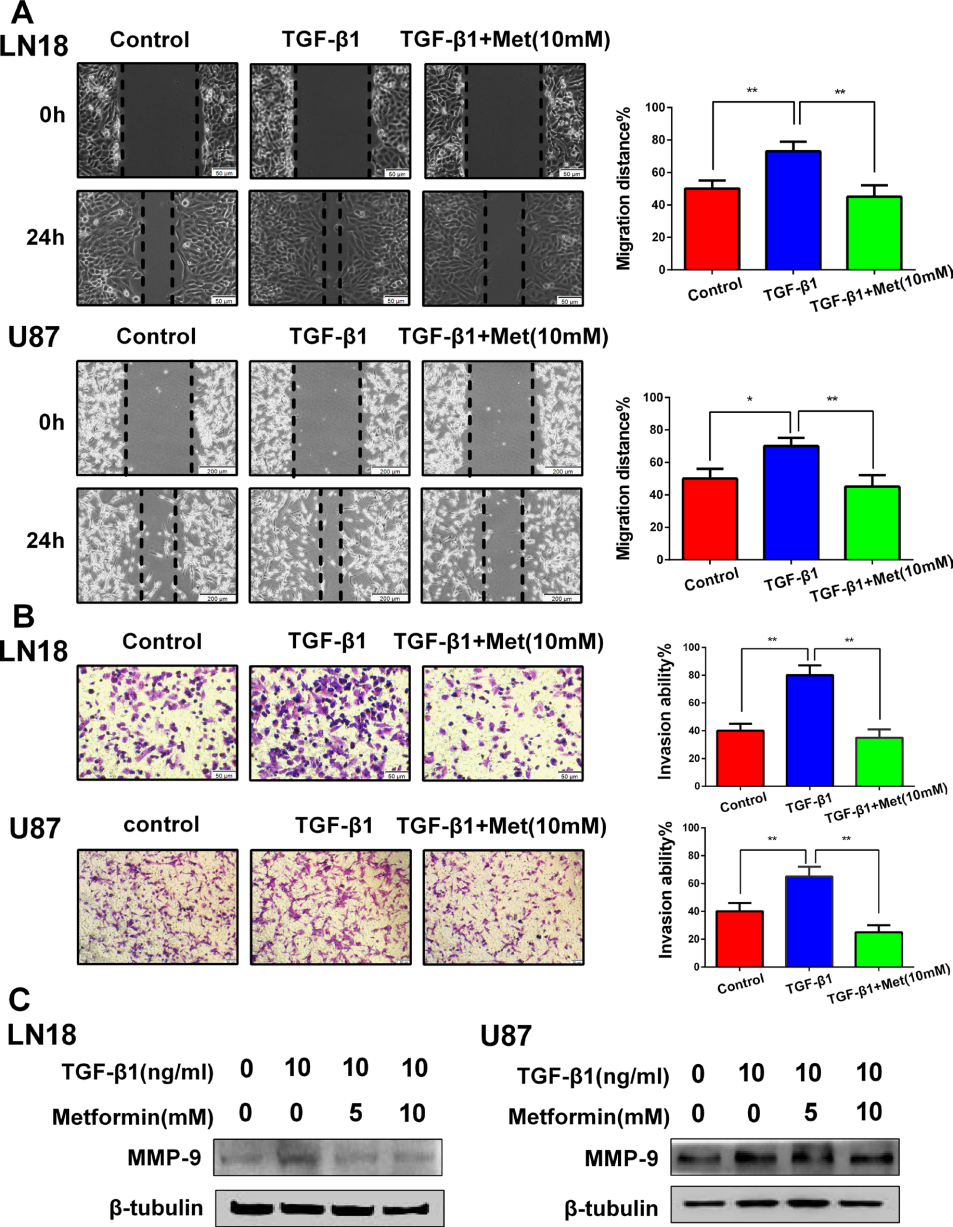
Metformin (Met) suppresses TGF-β1-induced cell migration and invasion (**A**) Representative wound-healing images show migratory capacity in LN18 (×100 magnification) and U87 (×40 magnification) cells following exposure to TGF-β1 (10 ng/ml) with or without metformin. Quantification histograms show the mean levels of migration distance observed in three random fields for each condition. (**B**) Representative Transwell photographs show invasion capacity in LN18 and U87 (×100 magnification) cells following exposure to TGF-β1 with or without metformin. Quantification histograms show the mean levels of the numbers of cells counted in five random fields on each filter for each condition. ^*^P < 0.05, ^**^P < 0.01 TGF-β1 treated group *vs.* control group; TGF-β1 treated group *vs.* TGF-β1 and Metformin (10 mM) treated group. (**C**) Western blot results of the expression levels of MMP-9 proteins in LN18 and U87 cells following treatment with TGF-β1 and metformin.

As anticipated, TGF-β1 promoted LN18 and U87 cells migration and invasion, whereas this tendency was blocked by metformin (Figure 3A and 3B). We further detected the invasion-related protein matrixmetalloproteinase-9 (MMP-9). The data showed that metformin significantly suppressed the TGF-β1-induced upregulation of MMP-9 expression (Figure 3C). These results suggest that the induction of TGF-β1 encourages migration and invasion, and this proces**s** can be dramatically blocked by metformin.

### Metformin reduces cancer stem-like properties generated by induction of TGF-β1

There is a tight link between TGF-β signal and cancer stem-like properties. Our results showed that induction of TGF-β1 resulted in the acquisition of self-renewal capacity and cancer stem-like expression pattern.

**Figure 4:**
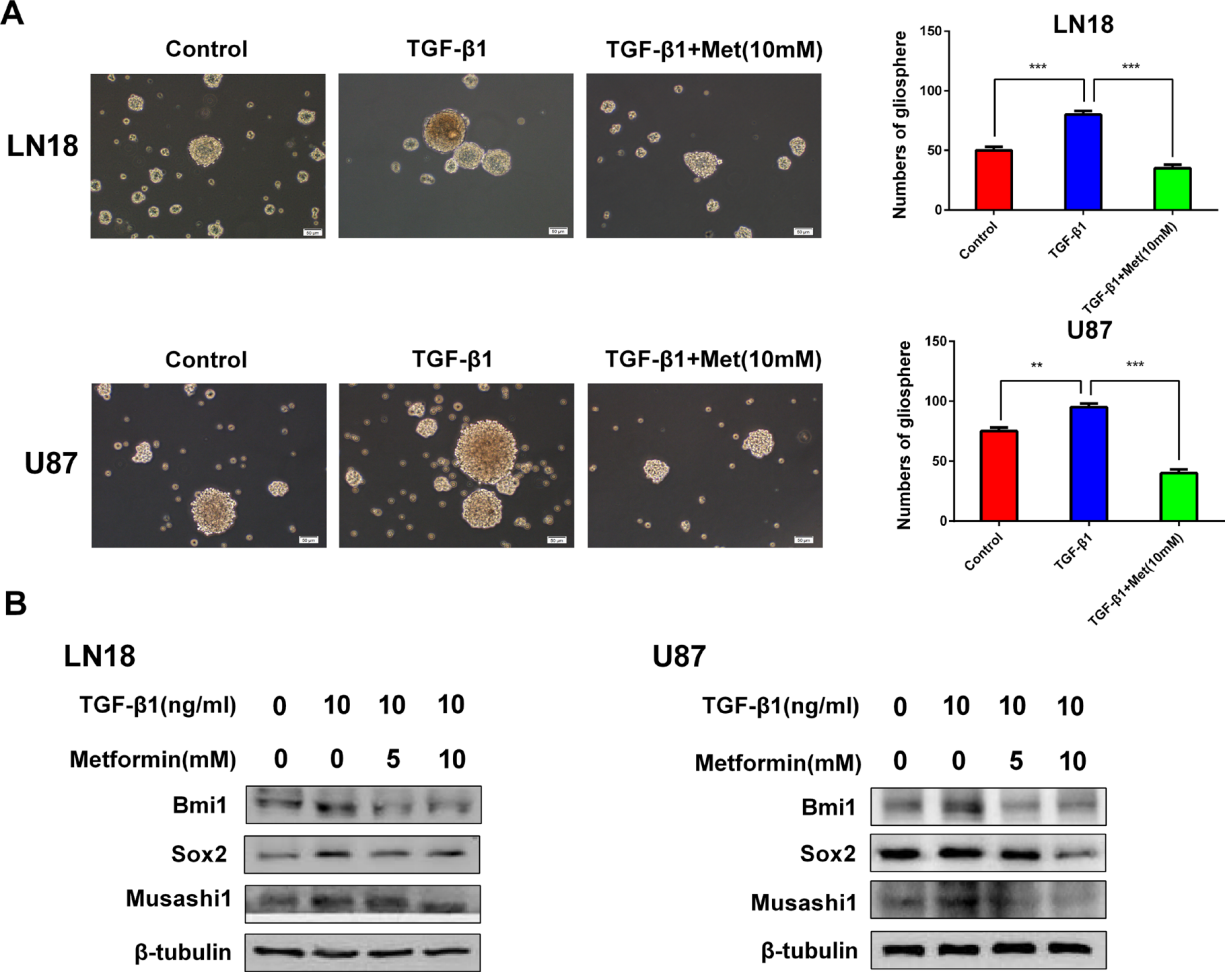
Metformin (Met) reduces cancer stem-like properties generated by induction of TGF-β1 (**A**) Metformin inhibited gliosphere formation in LN18 and U87 cells stimulated by exposure to TGF-β1 (10 ng/ml). Representative images of gliosphere were photographed under Olympus microscope (×100 magnification). Histograms show the numbers of gliosphere in different treatment groups. (**B**) Western blot results of inhibitory effect of metformin on stemness-related proteins stimulated by exposure to TGF-β1 in LN18 and U87 cells. ^**^P < 0.01, ^***^P < 0.001, TGF-β1 treated group *vs*. control group; TGF-β1 treated group *vs.* TGF-β1 and Metformin (10 mM) treated group.

The characteristic properties of CSCs are capable of forming tumorspheres in suspension cultures, this is a standard clonogenic assay for the detection of self-renewal of CSCs [17]. We investigated the effect of metformin on self-renewal capacity by tumor sphere formation assay. When cells were treated with TGF-β1, the efficiency of tumor sphere forming was obviously increased, whereas the sphere-forming ability was seriously impaired after exposure to metformin (Figure 4A).

The expression levels of CSCs markers, Bmi1, Sox2 and Musashi1, were obviously upregulated by induction of TGF-β1 (Figure 4B). Next, to determine the targeting effect of metformin on cancer stem-like properties, the expression levels of CSCs markers were analyzed. As anticipated, metformin specifically inhibited the expression levels of CSCs markers, Bmi1, Sox2 and Musashi1, in a dose-dependent manner (Figure 4B).

These findings strongly support that metformin is able to inhibit cancer stem-like properties generated by TGF-β1.

### Metformin inhibits AKT/mTOR pathway activated by TGF-β1

TGF-β1 can active several pathways including the Smad pathway and non-Smad pathway. As increasing evidence showing that AKT/mTOR pathway is often connected with proliferation and apoptosis of glioma cells, we chose to evaluate the potential role of TGF-β1 on the classical AKT/mTOR pathway.

**Figure 5:**
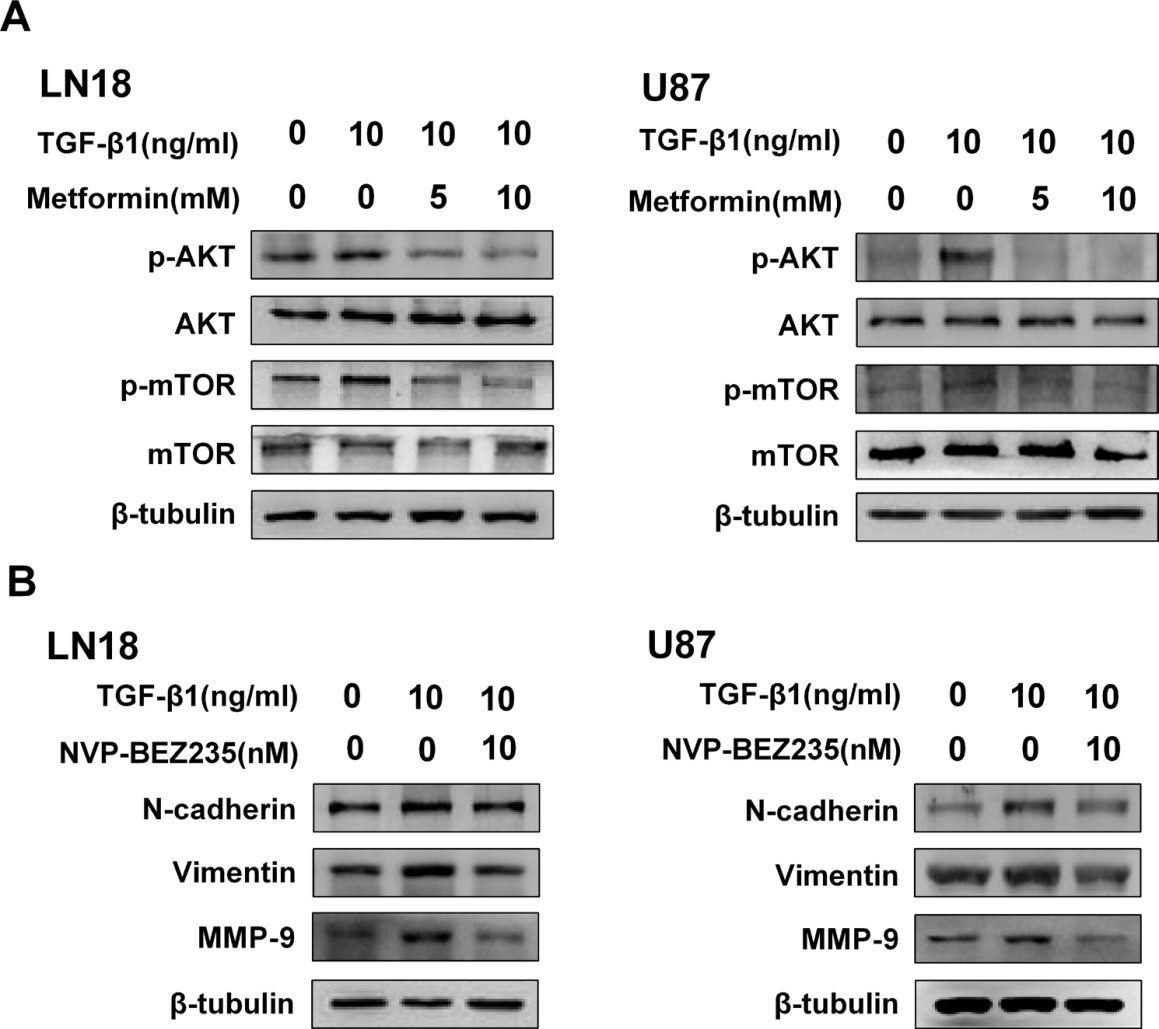
Metformin inhibits AKT/mTOR pathway activated by TGF-β1 (**A**) Western blot results of the inhibitory effect of metformin on TGF-β1-induced AKT/mTOR pathway. (**B**) Western blot results of the inhibitory effect of NVP-BEZ235 on N-cadherin, Vimentin and MMP-9 expression in LN18 and U87 cells.

We conducted an immunoblot analysis using specific antibodies to examine the phosphorylation status of AKT and the downstream molecular mTOR. As it is shown in Figure 5A, TGF-β1 activated phosphorylation status of AKT and its downstream molecular mTOR. And then, we further tested the inhibitory effect of metformin on this pathway. As expected, immunoblots analysis revealed phosphorylation status of AKT and mTOR was obviously downregulated by metformin treatment (Figure 5A).

To further show the role of AKT/mTOR pathway for inducing EMT-like process, we employed NVP-BEZ235, an imidazo [4,5-c] quinoline derivative that inhibits PI3K and mTOR kinase activity.

The direct role of AKT/mTOR pathway repression on the regulation of TGF-β1-induced EMT was determined by using NVP-BEZ235. Our result showed the inhibitor was effective in blocking TGF-β1 induced upregulation of N-cadherin and Vimentin in LN18 and U87 cells (Figure 5B). In addition, we went on testing the effect of NVP-BEZ235 on regulating the invasion-related protein marker of the GBM cells. As anticipated, the expression level of MMP-9 was also inhibited following treatment with NVP-BEZ235 (Figure 5B).

### ZEB1 is associated with the inhibitory effect of metformin on TGF-β1-induced EMT-like process in GBM cells

**Figure 6:**
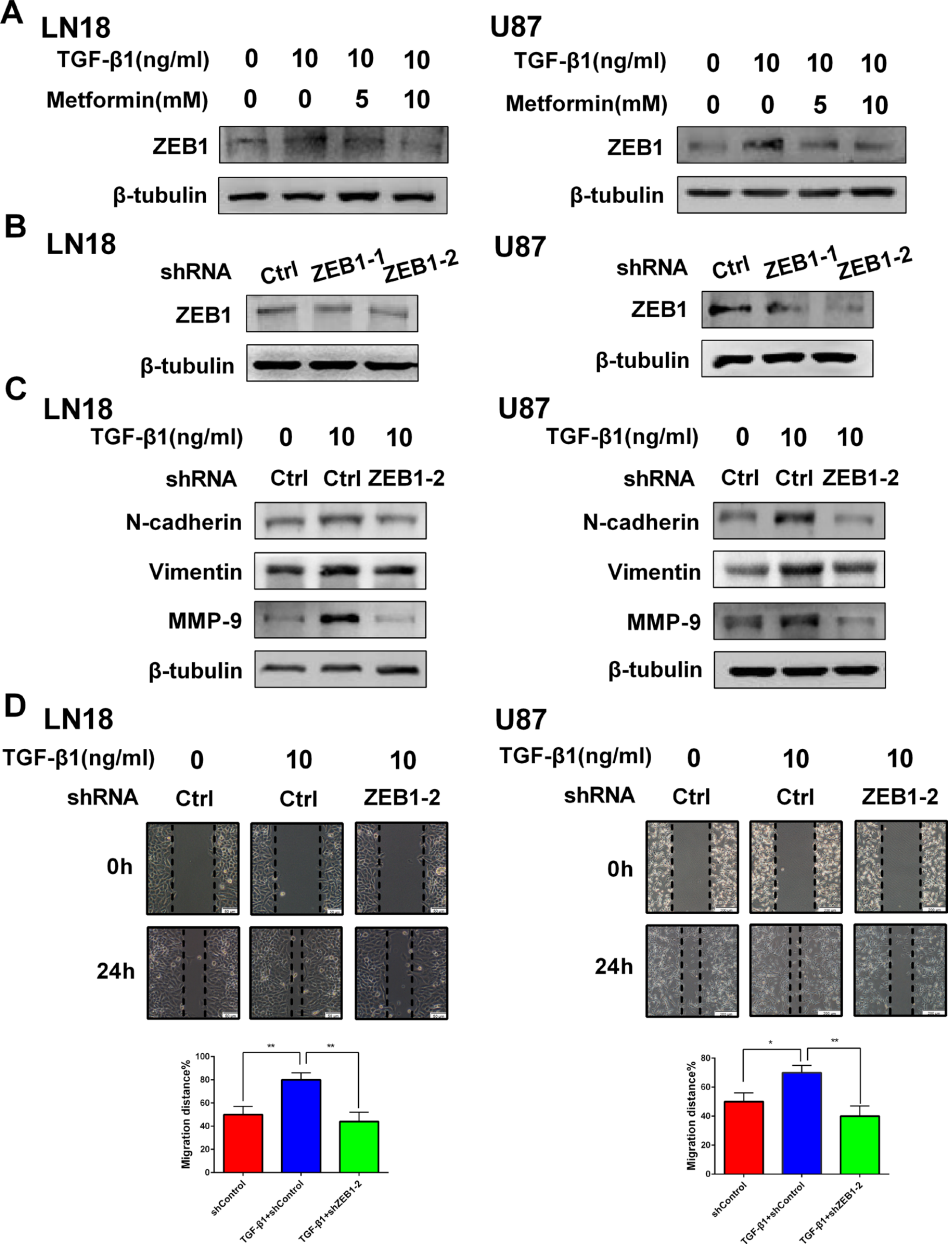
ZEB1 is associated with the inhibitory effect of metformin on TGF-β1 induced EMT-like process in GBM cells (**A**) Western blot results of the inhibitory effect of metformin on TGF-β1- induced ZEB1 expression in LN18 and U87 cells. (**B**) Western blot results of the effect of ZEB1 knockdown with two different shRNAs (shZEB1-1 and shZEB1-2) and control shRNA (shCtrl) on expression of ZEB1 in LN18 and U87 cells. (**C**) Western blot results of the effect of ZEB1 knockdown on expression of N-cadherin, Vimentin and MMP-9 induced by TGF-β1. (**D**) Representative wound-healing images show migratory capacity in LN18 (×100 magnification) and U87 (×40 magnification) cells following exposure to TGF-β1 (10 ng/ml) with or without ZEB1 knockdown. Quantification histograms show the mean level of migration distance observed in three random fields for each condition. ^*^P < 0.05, ^**^P < 0.01 TGF-β1+shCtrl group *vs.* shCtrl group; TGF-β1+shCtrl group *vs.* TGF-β1+shZEB1-2 group.

There are several factors connected with EMT. Among them, ZEB1 is the leading transcriptional factor involved in EMT [18]. In line with our anticipation, TGF-β1 activated the expression level of ZEB1. However, metformin could specially reduce the expression level of ZEB1 in a dose-dependent manner (Figure 6A), suggesting ZEB1 plays a role in modulating this EMT-like process.

To further validate ZEB1 is necessary for EMT-like process in GBM cells, we used short hairpin RNAs (shRNAs) to deplete ZEB1 (Figure 6B) and assessed the effect of ZEB1 inhibition on TGF-β1 stimulated EMT-like change in GBM cells. As a result, knockdown of ZEB1 in LN18 and U87 cells markedly suppressed TGF-β1-stimulated N-cadherin and Vimentin expression (Figure 6C).

Moreover, ZEB1 depletion attenuated TGF-β1-promoted cell migration properties of LN18 and U87 cells *in vitro* (Figure 6D). The expression level of MMP-9, an invasion-related protein, was also inhibited (Figure 6C).

**Figure 7:**
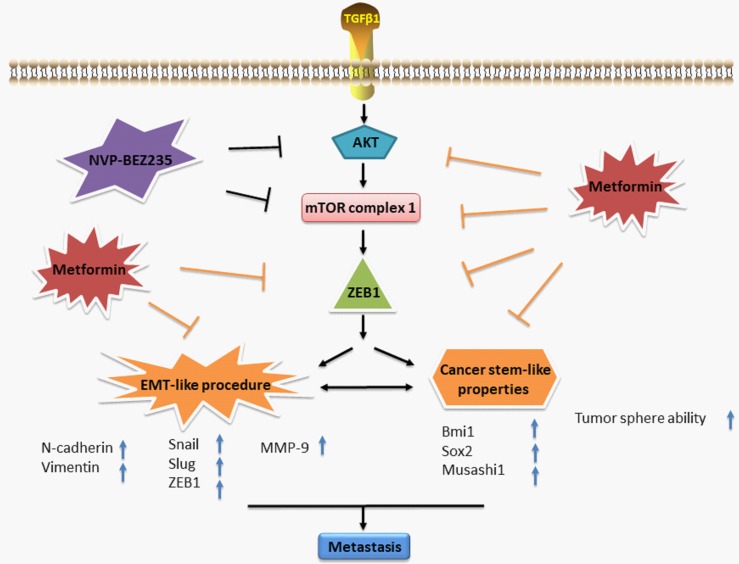
A working model illustrates the inhibitory effect of metformin on TGF-β1-induced EMT-like process in GBM cells

These data suggest that ZEB1 is involved with the inhibitory effect of metformin on EMT-like change in GBM cells. Figure 7 summarizes a possible working model describing the inhibitory effect of metformin on EMT-like process.

## DISCUSSION

Tumor invasion property is major cause responsible for treatment failure in the patients with glioblastoma. EMT has been shown to promote mesenchymal differentiation and migration in a number of human tumors including carcinomas of colon [19] and prostate [20]. However, it is still complicated and worth exploring in glioma cell lines [21]. To our knowledge, glioma cells can undergo an EMT-like process. The EMT-like process can vary from an intermediate phenotype to a phenotype that is less epithelial and more mesenchymal [6].

TGF-β1 regulates a variety of process in development and cancer and exerts many autocrine activities. In addition, TGF-β1 is also an important component of the GBM microenvironment [22, 23]. Taking these facts into consideration, we examined the inducing effect of TGF-β1 on EMT-like process in GBM cells. We found that treatment with TGF-β1 at 10 ng/ml was efficient enough to induce EMT-like change in GBM cells. We demonstrated that TGF-β1 treatment can not only induce the upregulation of the mesenchymal markers N-cadherin and Vimentin expression, but also increase expression levels of EMT-activating transcription factors, Snail, Slug and ZEB1, in GBM cells in a dose-dependent manner. Furthermore, treatment of TGF-β1 also led to the cell morphological changes and enhanced migratory ability in GBM cells. These findings indicate that TGF-β1 induces an EMT-like process in GBM cells.

EMT process is characterized by decreased expression of epithelial marker E-cadherin and increased expression of mesenchymal markers N-cadherin and Vimentin. Although E-cadherin is a major marker of EMT process in epithelial carcinomas, its expression appears to be limited in both GBM and normal brain samples [24]. Furthermore, E-to-N-cadherin switch has not been accepted as an essential marker for EMT in glioma [6]. So we didn't investigate the expression level of E-cadherin. Some of the transcription factors, such as Twist, Snail, Slug, and ZEB, which are actively involved in EMT process, also have a significant role in the EMT-metastasis linkage [25].

Since EMT-like process influences the prognosis of patients with GBM in a variety of aspects, it is in urgent to find suitable drugs targeted the specific procedure. Metformin seems to be able to target the EMT-like procedure. Recent evidence has indicated that metformin represses EMT in thyroid cancer cells [26] and cervical carcinoma cells [27]. However, whether metformin can also inhibit TGF-β1-induced EMT-like process in GBM cells remains to be unexplored. In this study, we found that metformin inhibited EMT-like process in GBM cells and changed these cells to a less mesenchymal phenotype as characterized by decreased expression of mesenchymal markers and important transcription factors. Furthermore, the wound-healing assay and Transwell assay showed that the inhibitory effect of metformin on EMT-like process was associated with repression of migratory and invasive abilities. Our results also showed that metformin treatment significantly inhibited TGF-β1-induced upregulation of invasion-related protein MMP-9 and expression levels of EMT-activating transcription factors Snail, Slug and ZEB1 in a dose-dependent manner. To our knowledge, this is the first time to demonstrate that metformin inhibits TGF-β1 mediated EMT-like process in GBM cells.

CSCs are a subpopulation of cells within heterogeneous tumors and are considered to play a crucial role in tumor progression, recurrence, metastasis and therapeutic resistance [28, 29]. Both CSCs and EMT have similar properties in tumor metastasis, recurrence and treatment resistance [30]. CSCs and EMT seem to be an axis of evil in cancer [31] and both CSCs and EMT may be the critical therapeutic target in cancer.

Previous study showed that TGF-β induces the self-renewal capacity of glioma stem cells (GSCs) and increases the expression levels of stemness-related factors Musashi1 and Sox2 [32]. To be continued, we next examined the inhibitory effect of metformin on cancer stem-like properties generated by exposure to TGF-β1. An essential property of CSCs is that the cells exhibit the capacity for self-renewal [17]. In the present study, the self-renewal capacity of GSCs was assessed with the secondary gliospheres (a tumorsphere from GSCs) formation assays *in vitro*. The formation of secondary gliospheres in control group indicated that GSCs had self-renewal capacity. Importantly, we found that treatment with TGF-β1 obviously enhanced the secondary gliosphere formation of GSCs, suggesting that TGF-β1 enhances self-renewal capacity of GSCs. As anticipated, we found that metformin significantly inhibited the enhanced the secondary gliosphere formation of GSCs, indicating that metformin can inhibit self-renewal capacity induced by TGF-β1. The stemness of GSCs has been linked to a number of transcription factors, such as Bmi1 [33], Sox2 [34]. Our finding was further supported by western blotting, which showed that expression of cancer stem markers, Bmi1, Sox2 and Musashi1, was markedly increased by treatment with TGF-β1 and decreased by treatment with metformin. Taken together, these data show that metformin can inhibit TGF-β1-induced cancer stem-like properties in GBM cells.

Then, we continued to explore the underline mechanism of this important transition. The occurrence of GBM is frequently associated with molecular changes in EGFR and AKT/mTOR pathway [35]. In addition, the Smad-independent signaling pathway induced by TGF-β1 leads to activation of AKT/mTOR or MAPK signaling [36]. Moreover, AKT activation is also correlated with the increased tumorigenicity, invasiveness and stemness [37].

In the present study, our results demonstrated that TGF-β1 enhanced phosphorylation of AKT and mTOR, suggesting that AKT/mTOR pathway is related to TGF-β1 mediated EMT-like change. Meanwhile we tested the effect of metformin on this pathway and the data showed that metformin decreased the phosphorylation of AKT and mTOR in a dose-dependent manner, indicating that the inhibitory effect of metformin is associated with AKT/mTOR pathway.

To further explore the effect of AKT/mTOR pathway, we inhibited AKT/mTOR activity using NVP-BEZ235, a dual PI3K-mTOR inhibitor. Recently, researchers have found that NVP-BEZ235 has not only an anti-tumor effect but also has the potential to inhibit EMT [38, 39]. In line with our anticipation, NVP-BEZ235 showed similar inhibitory effect by not only inhibiting mesenchymal markers but also decreasing the invasion-related protein MMP-9. Taking these facts into consideration, we demonstrated that AKT/mTOR pathway was closely correlated with the inhibitory effect of metformin on EMT-like process which was induced by TGF-β1 in GBM cells.

The transcription factor ZEB1 has important function in GBM progression, acting as a protumoral factor and is inversely correlated with survival in GBM patients [18]. Recently it is reported that metformin treatment transcriptionally downregulates the expression of EMT-related gene ZEB1 in breast cancer cells [40]. It is also showed that metformin may exert inhibitory effect of EMT in endometrial cancer cells through downregulating ZEB1 expression [41]. So, we further estimated that whether ZEB1 may influence the specific transition. We found that metformin inhibited the expression level of ZEB1 in a dose-dependent manner, indicating that metformin could exert inhibitory effect through downregulating ZEB1 expression. Furthermore, we knocked down the expression of ZEB1 and the data showed that the mesenchymal markers of N-cadherin and Vimentin were suppressed even though it is in the presence of TGF-β1. Knockdown of ZEB1 also reduced the migratory properties and decreased invasion-related protein marker MMP-9, reflecting an essential role of ZEB1 in controlling this transition process. These data show a new mechanism that ZEB1 mediates the inhibitory effect of metformin on TGF-β1-induced EMT-like change in GBM cells.

In conclusion, our results demonstrate that metformin effectively inhibits TGF-β1-induced EMT-like process and cancer stem-like properties in GBM cells by targeting AKT/mTOR/ZEB1 signaling pathway *in vitro.* These results indicate a potential clinical use of metformin in treatment of GBM and provide supporting evidence to warrant further clinical trial for metformin as a safe and potent anticancer drug.

## MATERIALS AND METHODS

### Reagents and antibodies

TGF-β1 was purchased from Peprotech (USA) and dissolved in 10 mM citric acid, pH 3.0, to a concentration of 0.1–1.0 mg/ml. Metformin was purchased from Sigma Chemical and dissolved in ultrapure water to obtain a stock concentration of 2000 mM. NVP-BEZ235, a dual PI3K/mTOR inhibitor, was purchased from Selleck Chemicals and dissolved in DMSO to obtain a stock concentration of 10 mM. All reagents were aliquoted and stored at −80°C and diluted to the desired final concentration in DMEM at the time of use. The final concentration of DMSO was less than 0.1% in all the cell cultures and did not exert any detectable effect on cell growth or cell death.

### Cell culture

The human glioblastoma cell lines LN18 and U87 were purchased from American Type Culture Collection (ATCC) and were authenticated by testing short tandem repeats (STR) using PureLink^®^ Genomic DNA Mini Kit in 2017. Both cell lines were cultured in DMEM media (Gibco, USA), supplemented with 10% fetal bovine serum (Hyclone, USA), Penicillin-Streptomycin (100 U/ml, Hyclone), glutamine (2 mM, Hyclone) in a humidified atmosphere of 5% CO_2_ at 37°C. The cells were dissociated using 0.25% trypsin and 0.02% EDTA solution and subcultured once in 2-3 days.

To generate GSCs, LN18 and U87 glioma cells were cultured in Neurobasal medium (NBM, Gibco) supplemented with N2 (1×, Gibco), B27 (1× , Gibco), glutaMAX (1×, Gibco), heparin (2 μg/ ml), recombinant human FGF-basic (b-FGF, 20 ng/ml, PeproTech), recombinant human epidermal growth factor (EGF, 20 ng/ml; PeproTech), Penicillin-Streptomycin (100 U/ml, Hyclone). The GSCs were cultured in 6-well plates in 5% CO_2_ incubator at 37°C with a medium change every 2–3 days. After gliospheres formed and reached 100–200 cells/sphere within 7 days, gliospheres were dissociated by Accutase (Sigma) and reseeded at a ratio of 1:2–1:3.

### Gliosphere formation assay

To test the effect of TGF-β1 or/and metformin on secondary gliosphere formation, after primary sphere formation was noted, the primary gliospheres were dissociated and plated in 96-well plates (5 × 10^3^ per ml per well) in NBM in the absence or presence of TGF-β1 or TGF-β1 + Met. Cultures were fed 0.02 ml of NBM every 2 days and photographed after 7 days using Olympus microscope.

### Cytotoxicity assay

For cytotoxicity assay, 3-(4,5-dimethylthiazol-2-yl)-2,5-diphenyltetrazolium bromide (MTT) was used. Cells (10^4^/per well) were cultured overnight in 96-well plates, then treated with TGF-β1 or metformin in indicated concentrations for 48 hours, and subjected to a standard MTT assay.

### Western blotting

The LN18 and U87 cells were cultured in 6-well plates and treated by drugs. The cells were washed with 4°C PBS twice, collected and lysed in RIPA Buffer. Then the lysate was centrifuged for 15 minutes and the supernatants were retained. The protein extracts were separated on 8%–12% SDS-PAGE and transferred to PVDF membranes. Membranes were blocked with 5% nonfat milk in 0.1% TBST for 1 hour and then incubated with primary antibodies against rabbit anti-N-cadherin, rabbit anti-Vimentin, rabbit anti-Snail, rabbit anti-Slug, rabbit anti-ZEB1, rabbit anti-p-AKT, rabbit anti-AKT, rabbit anti-p-mTOR, rabbit anti-mTOR, rabbit anti-MMP-9, rabbit anti-Bmi1, rabbit anti-Musashi1, rabbit anti-Sox2 (All of the above antibodies were procured from Cell Signaling Technology)overnight at 4°C, followed by incubation with secondary antibodies (1:5000 dilution, Boster, Wuhan, China). Equal lane loading was confirmed using a monoclonal antibody against β-tubulin (ORIGENE). After washing with the TBS-T buffer, the membranes were scanned with the Beijing Sage Creation Science Co., Ltd.

### Migration and invasion assays

The migratory capacity of cells was determined by wound-healing assays. 2 **×** 10^5^ cells were seeded on 6-well plates and upon confluency a scratch was made using pipette tip. The rate of migration distance was monitored at different time points under a microscope equipped with a camera.

The invasion capability of cells was assessed using 24-well matrigel-coated Transwell invasion assay. Cells (2.5 **×** 10^5^/ml) were suspended in 200 μl of serum-free medium and then plated on the top side of polycarbonate Transwell filter in the upper chamber of the BioCoat Invasion Chambers(BD). After incubated at 37°C for 48 hours, cells were removed from the upper chamber with cotton swabs.

Cells on the lower membrane surface were fixed in 4% formaldehyde and stained with 0.1% of crystal violet for 5 minutes. Randomly selected five fields of cells in each well were counted under a microscope.

### Transfection with shRNA plasmids

ZEB1 shRNAs (shZEB1-1 and shZEB1-2) and control shRNA (shCtrl) were purchased from Shanghai GeneChem Co. Ltd (Shanghai, China). Cells were transfected with ZEB1 shRNAs and control shRNAs using TransLipid HL Transfection Reagent (TransGen Biotech) according to the manufacturer's instructions.

### Statistical analysis

All experiments were performed intriplicate unless otherwise noted, and results were expressed as mean ± standard deviation. The *P*-values less than 0.05 were considered statistically significant. All of the analyses were performed with GraphPad Prism 6.0.

## Abbreviations

GBM: glioblastoma
EMT-like process: epithelial-to-mesenchymal transition-like process
CSCs: cancer stem cells
TGF-β: transforming growth factor-β
GSCs: glioma stem cells
MMP-9: matrixmetalloproteinase-9
shRNAs: short hairpin RNAs.
